# Comprehensive genetic and epigenetic analysis of sporadic meningioma for macro-mutations on 22q and micro-mutations within the *NF2 *locus

**DOI:** 10.1186/1471-2164-8-16

**Published:** 2007-01-12

**Authors:** Caisa M Hansson, Patrick G Buckley, Giedre Grigelioniene, Arkadiusz Piotrowski, Anders R Hellström, Kiran Mantripragada, Caroline Jarbo, Tiit Mathiesen, Jan P Dumanski

**Affiliations:** 1 Department of Genetics and Pathology, Rudbeck Laboratory, Uppsala University, 751 85 Uppsala, Sweden; 2 Department of Neurosurgery, Karolinska University Hospital, Solna, SE-171 76 Stockholm, Sweden; 3 University of Alabama at Birmingham, 1530 3rd. Ave. S., Kaul 420, Birmingham, AL 35294-0024, USA

## Abstract

**Background:**

Meningiomas are the most common intracranial neoplasias, representing a clinically and histopathologically heterogeneous group of tumors. The neurofibromatosis type 2 (*NF2*) tumor suppressor is the only gene known to be frequently involved in early development of meningiomas. The objective of this study was to identify genetic and/or epigenetic factors contributing to the development of these tumors. A large set of sporadic meningiomas were analyzed for presence of 22q macro-mutations using array-CGH in order to identify tumors carrying gene dosage aberrations not encompassing *NF2*. The *NF2 *locus was also comprehensively studied for point mutations within coding and conserved non-coding sequences. Furthermore, CpG methylation within the *NF2 *promoter region was thoroughly analyzed.

**Results:**

Monosomy 22 was the predominant finding, detected in 47% of meningiomas. Thirteen percent of the tumors contained interstitial/terminal deletions and gains, present singly or in combinations. We defined at least two minimal overlapping regions outside the *NF2 *locus that are small enough (~550 kb and ~250 kb) to allow analysis of a limited number of candidate genes. Bialleinactivationo the *NF2 *gne was detected in 36% of meningiomas. Among the monosomy 22 cases, no additional *NF2 *mutations could be identified in 35% (17 out of 49) of tumors. Furthermore, the majority of tumors (9 out of 12) with interstitial/terminal deletions did not have any detectable *NF2 *mutations. Methylation within the *NF2 *promoter region was only identified at a single CpG site in one tumor sample.

**Conclusion:**

We confirmed previous findings of pronounced differences in mutation frequency between different histopathological subtypes. There is a higher frequency of biallelic *NF2 *inactivation in fibroblastic (52%) compared to meningothelial (18%) tumors. The presence of macro-mutations on 22q also shows marked differences between fibroblastic (86%) and meningothelial (39%) subtypes. Thus, inactivation of *NF2*, often combined with the presence of macro-mutation on 22q, is likely not as important for the development of the meningothelial subtype, as opposed to the fibroblastic form. Analysis of 40 CpG sites distributed within 750 bp of the promoter region suggests that *NF2 *promoter methylation does not play a major role in meningioma development.

## Background

Meningiomas are the most frequently occurring intracranial tumors. Clinically treated meningiomas comprise approximately 13–26% of all primary brain tumors [1]. Epidemiological studies indicate that ~90% of all meningiomas are asymptomatic [2]. The majority are benign, slowly growing, solitary and sporadic tumors, but atypical or malignant meningioma constitutes approximately 10% of all cases. Meningiomas are thought to be derived from the arachnoid cap cells of the leptomeninges, which is the covering of the brain and the spinal cord [1,3]. They usually occur in the cranial meninges, but approximately 10% of tumors develop in the meninges covering the spine. From histopathological and clinical points of view, meningiomas are a heterogeneous group of tumors, which are classified into 15 histopathological types with WHO malignancy grades I–III [4].

Meningiomas was the first solid tumor to be characterized as containing a specific genetic aberration, which is monosomy 22 [5]. Since then, loss of genetic material from chromosome 22 has been the most consistent aberration, observed in up to 70% of tumors [6,7]. Meningiomas are closely associated with the genetic syndrome neurofibromatosis type-2 (NF2). Approximately 50% of NF2 patients suffer from meningiomas, making them the second most frequent neoplasm associated with this tumor syndrome [8-10]. In 1993, the neurofibromatosis type 2 (*NF2*) tumor suppressor gene was characterized from chromosome 22 [11,12]. Sporadic meningiomas were therefore screened for mutations in the *NF2 *gene, which was found to be frequently inactivated. There is, however, a wide discrepancy in the reported mutation frequency (20–60%) [7,13-18]. In fact, it has been shown that meningiomas may be grouped into tumors with and without mutations in the *NF2 *gene. These groups can also be correlated with histopathological subtypes [15,17]. Tumors with mutations in the *NF2 *gene are often of the transitional or fibrous subtype, commonly with allelic losses on chromosome 22. Tumors without *NF2 *gene mutations are predominantly of the meningothelial subtype, and allelic losses on chromosome 22 are less commonly observed in this subtype. Furthermore, the meningothelial meningiomas are more often located in the anterior parts of the skull base [19]. Transcriptional silencing of the *NF2 *tumor suppressor by CpG methylation in meningiomas was recently tested by two research groups, but the results were conflicting. Van Tilborg *et al*. reported methylation in 1 out of 21 tumors, whereas Lomas and colleagues reported a higher methylation frequency (23 out of 88 cases) [20,21].

Despite almost 40 years of cytogenetic and molecular genetic research, the *NF2 *tumor suppressor is still the only specific gene which has been shown to be frequently involved early in the development of meningiomas. There is, however, a general consensus that other not yet characterized loci are important for the development and/or progression of these tumors [22-27]. Considering the clinical and histopathological heterogeneity of meningiomas, it is possible that alternative genetic mechanisms underlay its tumorigenesis. There are reports of the loss of chromosome 22 as the primary consistent aberration in meningiomas, in the absence of mutations in the *NF2 *gene. Moreover, young patients with constitutional ring chromosomes and multifocal meningiomas have been characterized. This highlights the possibility of an alternative genetic etiology related to other genes located on chromosome 22 [23,28-30]. In addition, other sites in the genome have been implicated in meningioma development or progression [23]. Among these, aberrations of chromosome 1 have been established as the second major genetic factor for the initiation/progression of meningiomas and correlate with increased morbidity [31,32]. Furthermore, the *NF2 *gene has also been excluded by linkage analysis in one family with multiple meningothelial meningiomas and ependymomas [33]. These various reports suggest that there exist additional loci contributing to the formation of meningiomas.

The rationale of this study was to perform a multilevel analysis of mutations that include gene dosage aberrations on chromosome 22, analysis of point mutations in the *NF2 *locus, and profiling of the methylation status within the *NF2 *gene promoter. We also intended to relate our findings from chromosome 22 to the high resolution DNA copy number variation in meningiomas of chromosome 1, which was recently reported [34].

## Results and discussion

We analyzed a comprehensive series of sporadic meningiomas on multiple levels, i.e. for presence of "macro-mutations" involving large scale aberrations of 22q using an array-based approach and for "micro-mutations" within the *NF2 *tumor suppressor gene. The latter involved analysis of point mutations within the mRNA encoding part of this gene and within the conserved non-genic (CNGs) sequences, which we have previously defined [35]. We also comprehensively studied the promoter region of the *NF2 *gene for CpG methylation.

### Chromosome 22 DNA copy number profiling using array-CGH

We studied 126 sporadic meningioma samples, composing a representative series of tumors operated on at a single hospital in Sweden. All tumor samples studied were analyzed previously using a lower resolution approach [7,22,23,31,36]. The main rationale in this part of the project was to identify tumors possessing gene dosage aberrations of 22q which did not involve the *NF2 *locus. We carried out DNA copy number profiling using array-CGH on a genomic microarray covering chromosome 22. This array, containing 480 measurement points across the sequenced part of this autosome, was developed and validated previously [37]. Among the samples used for validation of this array was meningioma T24 DNA, which is a well characterized tumor containing a large terminal deletion of 22q and an additional approximately 150 kb biallelic deletion involving *EWSR1*, *GAR22*, *RRP22*, *AP1B1*, *RFPL1 *and *NEFH *genes [37,38]. On average, each array-CGH experiment generated ~400 positively scored data points from 22q.

**Figure 1:**
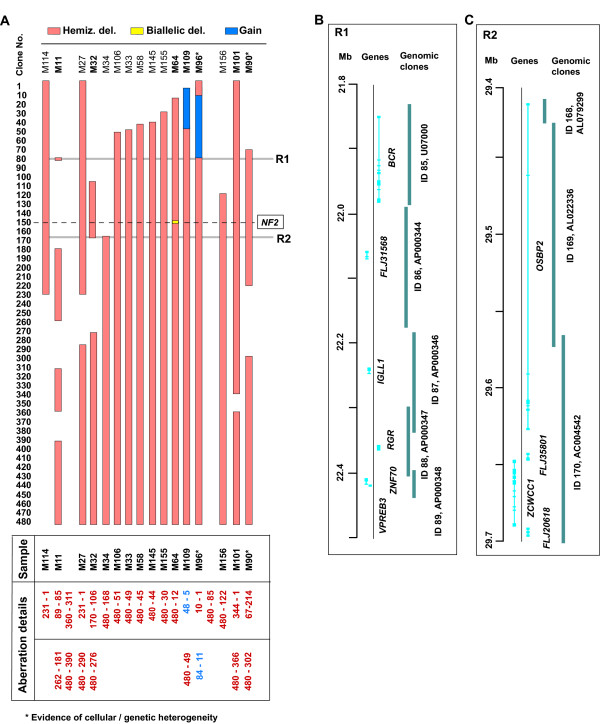
**Summary of array-CGH profiles for chromosome 22 insporadic meningioma**. **A) **Detailed summary of all complex aberrations. Deletions and gains are mapped according to the clones present on the chromosome 22 minimal tiling path array. Chromosome 22 clones are grouped in bins of 10 along the y-axis. The panel of tumors which displayed complex aberrations of chromosome 22 is shown from left to right. The red, yellow and blue boxes denote hemizygous deletion, biallelic deletion and gain, respectively. The horizontal dashed line displays the approximate position of the *NF2 *gene in relation to the measurement points on the chromosome 22 minimal tiling path array. The table below details the clone ID's at which the deletion/gain starts and finishes. Smallest regions of overlapping deletions are highlighted with grey lines (R1 and R2) and genes present within these regions are shown to the left. Tumor names highlighted in bold are displayed in more detail in other figures. **B) **Detailed view of candidate region 1 (R1) with gene and clone information presented according to the positions on chromosome 22 sequence (UCSC Homo sapiens Genome Browser, assembly July 2003). **C) **Detailed view of candidate region 2 (R2) with gene and clone information presented according to the positions on chromosome 22 sequence (UCSC Homo sapiens Genome Browser, assembly July 2003).

**Figure 2:**
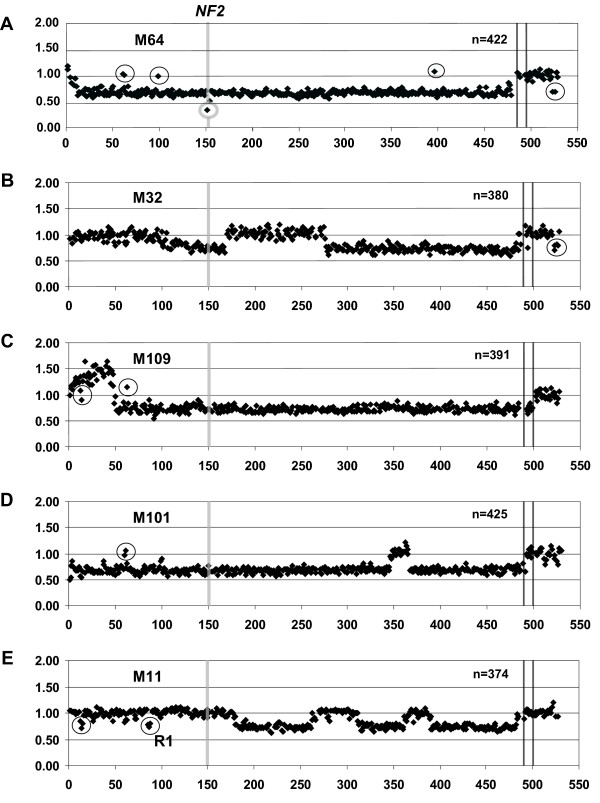
**Array-CGH profiles of chromosome 22 in tumors displaying complex aberrations**. The chromosome 22 array-CGH plots consist of 480 chromosome 22 clones ordered from centromere to telomere and 9 chromosome X controls between the vertical lines, as well as 31 non-chromosome 22 controls plotted on the right hand side. The y-axis displays the raw fluorescent intensity ratios for each clone on the genomic array. The position of the *NF2 *gene is highlighted with the vertical grey bar. **A) **Array-CGH profile of case M64 displaying a single copy loss of the majority of the chromosome with a biallelic deletion encompassing a single measurement point (clone ID 151) [GenBank:AC005529], specifically targeting the *NF2 *gene (highlighted by a grey circle). Encircled clones centromeric of *NF2 *display fluorescent ratio consistent with two copies; clone IDs 61–63 [GenBank:D87000–D87003] (previously reported Copy Number Polymorphism, CNP) and clone IDs 99–100 [GenBank:AL049759–AL050312]. Clone ID 400 [GenBank:Z84478] positioned towards the telomere, also exhibit two gene copies (highlighted by a circle). Control clone derived from chromosome 10 encompassing *SUFU1B *gene presents with one copy loss (highlighted by a circle). **B) **Array-CGH profile of case M32 displaying an interstitial deletion encompassing the *NF2 *gene and a terminal deletion. Clones spanning the *DAL-1 *gene on chromosome 18 and clones spanning the *PTCH1B *locus on chromosome 9 display a fluorescent ratio consistent with a one copy loss are highlighted by a circle. **C) **Case M109 displaying a gain spanning clone IDs 5–48 [Genbank:AC006548–AC007957] followed by a large terminal deletion covering clones 49–480 [Genbank:AC009516–AC002055]. Two CNPs are positioned at clone IDs 13–15 and 63 [Genbank: AC008079–AC008132 and D87003] and are indicated by circles. **D) **Meningioma M101 displaying a large one copy loss for the majority of the chromosome apart from a region encompassing clone IDs 348–366 [GenBank:AL080242–Z82176] and a CNP spanning clone IDs 60–61 [GenBank:D88269–D87000]. **E) **Detection of multiple interstitial deletions outside the *NF2 *region, spanning clone IDs 85–89 (R1, highlighted by a circle), 181–262 and 311–360 [GenBank: U07000–AP000348, AC005004–Z69907 and AL022311–Z83851] and a terminal deletion covering clone IDs 390–480 [GenBank:Z93244–AC002055] in case M11. A CNP spanning clone IDs 14–15 [GenBank:AC008132–AC008103] is highlighted by a circle.

**Figure 3:**
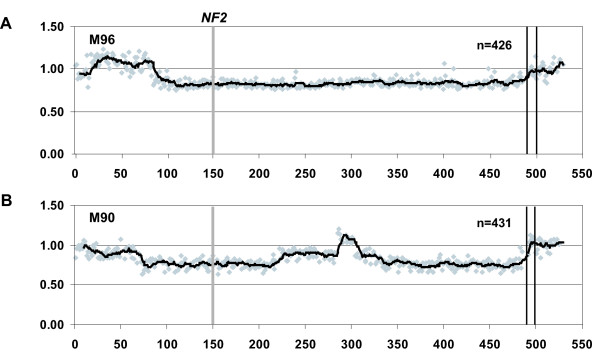
**Detection of cellular or genetic heterogeneity in meningioma samples**. The array-CGH plots follow the same general scheme as for Figure 3. However, the data points are shaded in grey and a trend line (with a moving average of 10 data points) is shown, which highlights the difference in fluorescent ratio levels along the chromosome. **A**) Identification of genetic heterogeneity in meningioma sample M96 which displays 3 different fluorescent ratio values across chromosome 22. The clone IDs and **A**verage **N**ormalized **I**nter **L**ocus **F**luorescence **R**atio (ANILFR) for each of these are 1–15, 0.95; 16–84, 1.1 and 85–480, 0.83, respectively. **B**) Array-CGH profile of a complex set of aberrations on chromosome 22, which suggest genetic/cellular heterogeneity within the tumor M90. The regional shifts in fluorescent ratio across chromosome 22 are as summarized with the clone IDs and their ANILFR value; 1–23, 0.95; 24–66, 0.90; 67–214, 0.76; 215–284, 0.88; 285–303, 1.09; 304–326, 0.87; 327–480, 0.76. The non-chromosome 22 autosomal controls have an ANILFR value of 1.00, since this value was used to normalize all values across the genomic array.

Table 1 summarizes results obtained from DNA copy number profiling of chromosome 22 and other results, as well as relevant clinical information pertaining to the studied patients. The predominant profile detected in 59 tumors was monosomy 22, which accounted for 47% of meningiomas, and this result is consistent with previous studies [7,22,23,31,36]. The biological consequence of this very common aberration for the affected tumor cells is one copy loss of all genes from 22q. The second category comprised of16 tumors (13%) that were affected by terminal deletions, interstitial deletions and gains, present singly or in combinations (Figure 1A). This number is also consistent with previous analysis using a loss of heterozygosity (LOH) approach [7,22,23,31,36]. However, due to a much higher density of data points used in the current analysis, we were able to detect additional aberrations as well as exactly define the breakpoints of all rearrangements in samples which were characterized previously using LOH. Figure 1A shows all 16 cases in detail, and Figs. 2 and 3 display array-CGH profiles of 7 tumors providing new information. Tumor M64 (Figs. 1A and 2A) disclosed a previously unknown biallelic deletion affecting the *NF2 *gene. This was the only locus affected by a homozygous deletion in the studied series of tumors, which confirms the importance of the *NF2 *gene in meningioma development. This fibroblastic meningioma has an overall 22q profile consistent with a very large (> 30 Mb) terminal deletion of the long arm of chromosome 22. The second deletion affects only one clone (ID 151) [GenBank: AC005529] and specifically targets the *NF2 *gene, thus inactivating its second allele in tumor cells. Another interesting example is fibroblastic meningioma M11, since it displays a terminal and multiple interstitial deletions, all of which are located outside the *NF2 *locus (Figs. 1A and 2E). Three of these deletions, located towards the telomere of 22q, were previously characterized [39]. However, current analysis revealed an additional, smaller (~550 kb) deletion, encompassing 5 contiguous clones (ID 85–89) [GenBank: U07000–AP000348], located on the centromeric side of the *NF2 *locus (Figures 1A, 1B and 2E). This tumor is intriguing since no additional genetic/epigenetic aberration in the *NF2 *gene could be detected in this sample (see below). The vast majority of copy number aberrations of 22q in meningiomas point to inactivation or a decreased gene dosage. However, in two tumors (M109 and M96, Figs 1A, 2C and 3A) we were able to detect a low copy number gain. For instance, tumor M109 displays a combination of low copy number gain in the centromeric part of 22q and a large terminal deletion (Figure 2A). A minimum overlapping region of gain is approximately 3.8 Mb (clone ID 11–48) [GenBank: AC006285 – AC007957] and is located in 22q11. The importance of these gains for meningioma tumorigenesis is difficult to judge, as they are not frequent events. One possible explanation is that these gains reflect cell-specific chromosomal instability of 22q11, since this region is rich in intra-chromosomal segmental duplications and is prone to rearrangements leading to several genetic disorders.

All samples displaying aberrations other than monosomy 22 are graphically summarized in Fig 1A. This analysis enables the discrimination of several minimal overlapping deleted regions which should be studied further. Two of these regions, named as R1 (~550 kb) and R2 (~250 kb) are small enough to allow for detailed analysis of a limited number of candidate genes (Figs. 1B and 1C). The *breakpoint cluster region (BCR) *gene is one interesting candidate located in R1. This gene is located at the site of the chromosome 22 breakpoint generating the Philadelphia chromosome (9;22) translocation that creates the *BCR/ABL *fusion gene found in chronic myeloid leukemia and acute myeloid leukemia. The normal *BCR *gene was reported to have a serine/threonine kinase activity [40]. Another candidate gene located within R1 is the *RGR *oncogene. This gene was originally identified as a fusion protein which can promote tumor development in nude mice, but has also displayed oncogenic properties when not in the fusion form [41]. The *RGR *gene has frequently been found to be altered in human T-cell malignancies [42]. The zinc finger protein encoding gene *ZCWCC1*, located within R2, may also be a candidate for further investigation. The relative weakness of the R1 and R2 regions is that they are defined by rare cases with small deletions. There is therefore a possibility that the latter chromosome 22 aberrations are neutral "passenger mutations", with little consequence to tumorigenesis. Yet another apparent, large candidate region is located towards the telomere of 22q, starting from clone ID 390. This segment is affected by hemizygous deletion in all but one (M114) of the studied tumors which disclosed gene copy number imbalances of chromosome 22.

The third major category of tumors (51 cases, 40%) was composed of samples that did not display detectable changes in gene dosage on 22q (Table 1, "**N**o **A**berration **D**etected"; NAD) despite the high average resolution (~75 kb per data point). There may be several possible explanations for these results. One possibility is that additional genes on 22q (other than *NF2 *tumor suppressor) are important for meningioma development and these are mutated by alternative mechanisms, such as point mutation or epigenetic silencing. Another explanation might be that this subset of samples involves an alternative tumorigenic pthway which does not involve chromosome 22-located genes.

The current version of the chromosome 22 array has been supplemented with additional non- 22q-derived genomic clones from other chromosomes, which encompass genes (e.g. *PTCH2A*, *PTCH1B*, *SUFU *and *DAL1 *genes) of potential interest in the fields of meningiomas and brain tumors in general. The complete list of these additional clones is provided in additional file 1. Table 1 shows the samples in which loss of DNA copy number was detected for some of these loci. The highest rate of loss detected was at the *PTCH2A *locus on 1p34.1, which displayed a rate of loss of 18%. The other loci, namely *DAL-1, PTCH1B*, *SUFU1A *and *SUFU1B*, displayed a level of DNA copy number loss of 6%, 5%, 6% and 6%, respectively. We also detected DNA copy number losses in a number of non-chromosome 22-derived control clones. The most frequently deleted control clones were those derived from chromosomes 1 and 3, suggesting the likely importance of other regions of the genome in meningioma development/progression.

### Mutation analysis of *NF2 *exons and evolutionarily conserved non-genic sequences (CNGs)

**Figure 4:**
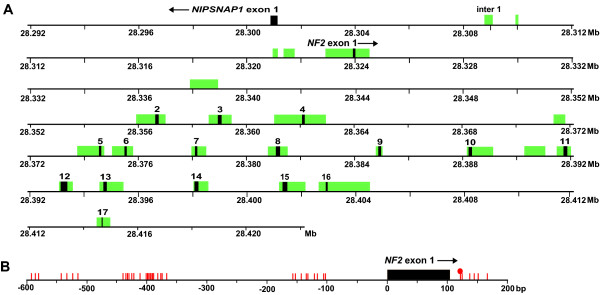
**Overview of the *NF2 *locus analyzed for point mutations and methylation of CpG dinucleotides**. **A) **Illustration of the *NF2 *gene displaying exons (black boxes) and conserved non-genic sequences (CNGs) (green boxes) that were analyzed for point mutations. Gene orientation is displayed with an arrow next to the gene name. The scale is according to sequence position on chromosome 22 in million base pairs (Mb) (UCSC Homo sapiens Genome Browser, assembly July 2003). **B) **Schematic view of the CpG sites in the 5' part of the *NF2 *gene that were analyzed for methylation. The 40 sequenced CpG sites were located between positions -591 and +166 bp (position +1 refers to transcriptional start site). A single methylated CpG site was detected in tumor M104 at position +120 in ~40% of DNA molecules (displayed as a red filled circle).

Inactivating micro-mutations in the *NF2 *tumor suppressor gene are found in meningiomas of all histopathological subtypes and malignancy grades and are thought to be an early event in tumorigenesis [43]. However, large differences (20–60%) of mutation frequency in sporadic meningiomas were previously reported [7,13-18]. Our rationale was to reassess the mutation frequency of *NF2*, especially when combined with very high resolution array-CGH analysis of these tumors. The rationale was also to test the hypothesis that mutations might be located in evolutionarily well conserved non-genic sequences within introns or in the 5' promoter region of the *NF2 *gene, in addition to mutations in the exons. Comparisons of the orthologous genomic sequence from the *NF2 *locus derived from five species (human, baboon, mouse, rat and pufferfish) were previously performed in order to identify CNGs that might be of functional importance for the *NF2 *gene [35]. The exonic sequence of the *NF2 *gene constitutes ~2.7% of this locus. When applying a threshold of 70% sequence identity over >100 bp of gap-free alignment in comparisons between human and mouse, the resulting level of extra-exonic conservation was 3.8%. We selected CNGs for mutational analysis that displayed a similar level of evolutionarily conservation as the protein coding sequence. In total, 17.5 kb was sequenced in 100 tumors, which included all 17 exons, the highly conserved exon flanking sequences and seven CNGs not contiguous with exons (Figure 4A). Four of the CNGs were positioned upstream of exon 1 and the remaining three CNGs were located in introns 1, 4 and 10.

Thirty-nine alterations were identified in 100 analyzed samples (Tables 1, 2 and 3). Fifteen mutations caused frame-shifts, twelve altered the acceptor- or donor-splice sites, nine were nonsense, two were missense and one mutation occurred in the *inter1 *CNG. None of the identified mutations were present in the corresponding peripheral blood DNA. Thirty-five of these mutations were accompanied by a macro-mutation on the other allele, either through monosomy or terminal/interstitial deletions. Overall, we could detect mutations consistent with biallelic inactivation of the *NF2 *tumor suppressor gene in 36% of meningiomas (Table 3). However, in three cases (M46, M115 and M87) no second inactivating mutation could be identified. Considering what is known to date about the *NF2 *gene mutational spectrum in different tumors, it can be assumed that at least two of the latter three mutations (M46 – aberrant splicing and M115 – nonsense mutation) are pathogenic. These types of mutations are, however, usually combined with the second inactivating hit on the remaining normal allele of the gene [13,44-47]. The third aberration is a missense mutation (tumor M87, exon 4, 436 G>A, Val-146-Ile, Table 1), which is possibly introducing a dysfunction to the N-terminal FERM domain (residues 1–302 of the protein, plasma membrane binding domain) of the protein. Merlin/schwannomin provides a link between the cytoskeleton and plasma membrane and interacts with the actin cytoskeleton through binding sites in its FERM domain [48,49]. Thus, there exists a possibility of this missense mutation exerting a sufficiently strong dominant negative effect on the protein function, which does not necessitate the presence of the second hit *NF2 *gene inactivating event in this tumor. The three tumors discussed above (M46, M115 and M87) allow us to estimate our false negative detection rate of mutations in the *NF2 *gene. We can speculate that, in at least two and a maximum of three of the studied tumors, we could expect the second hit *NF2 *gene mutation to be present, but we were unable to detect it. Assuming the above reasoning to be correct, our false negative rate of micro-mutation detection is between 5.1 – 7.6%.

In case M22 we identified two tumor-specific micro-mutations, one mutation causing a frame shift in exon 13 introducing a premature stop codon and the second being a mutation in the *inter1 *CNG element. *Inter1 *is situated approximately 15 kb upstream of the *NF2 *gene (Figure 4A). It displays sequence conservation between human and pufferfish, which are evolutionarily very distant species [35], suggesting that *inter1 *may possess functional properties. Details on the exact position of the *inter1 *mutation on chromosome the 22 sequence are shown in Table 1. The tumor-specific mutation in *inter1 *CNG is one of three changes present in M22, as this tumor also contains monosomy 22. Thus, we may speculate that three hits are affecting the *NF2 *gene. An alternative explanation is that one of these mutations has little consequence to the normal function of the *NF2 *gene.

### Methylation profiling of the *NF2 *promoter region by bisulfite modification followed by pyrosequencing

Methylation of promoter associated CpG-islands is known as a possible mechanism for inactivation of tumor suppressor genes in a variety of human cancers. It has also been suggested as a mechanism of repressing transcription of the *NF2 *gene in schwannomas [50] and in meningiomas [21]. Methylation of other cancer related genes (*GSTP1*, *THBS1*, *CDKN2A*, *CDH1*, *BRCA1*, *p14*^*ARF *^and *RB1*) has also been reported to be associated with malignant progression of meningiomas [20,51,52]. We used an established method of bisulfite treatment of tumor DNA followed by Pyrosequencing (Biotage, Uppsala, Sweden) which allows detection and semi-quantitative estimation of the degree of methylation at the selected CpGs [53,54]. Two overlapping PCR products of 339 bp and 550 bp covered ~750 bp of the upstream promoter region, the first exon and approximately 70 bp of intron 1, immediately downstream of the first exon (Figure 4B). These two products were Pyrosequenced with 8 sequencing primers. In total, 40 CpG sites were sequenced, between positions -591 and +166 bp (position +1 refers to transcriptional start site). This analysis allowed interrogation of up to 11 CpG sites per assay, distributed over 79 bp and with a maximum of 60 dispensations of nucleotides (additional file 2). Methylation was identified in only one tumor sample (M104), at a single CpG site at position +120, in ~40% of DNA molecules in this tumor (Figure 4B). Kino *et al*. reported a 70 bp region (-591 to -522) essential for *NF2 *expression in schwannomas [50]. Three CpG sites within this region (at positions -591, -586 and -581) appeared to be of particular importance for silencing of the *NF2 *gene upon methylation in schwannomas. We paid particular attention to these CpG sites but no methylation was detected. In summary, upon comprehensive analysis of 40 CpG sites, we conclude that methylation does not play a major role in the silencing of the *NF2 *gene in meningiomas. These results are in accordance with a report by van Tilborg *et al *[20] although contradicting the results from Lomas *et al*. [21].

### Summary of macro- and micro-mutations on chromosomes 22 and 1 in meningiomas

We observed pronounced differences of the *NF2 *gene mutation frequency between different histopathological subtypes of meningiomas (Table 3). There was a clear trend towards an increased frequency of the biallelic *NF2 *gene inactivation in meningothelial, transitional and fibroblastic subtypes which were 6 out of 33 tumors (18%), 12 out of 31 tumors (39%) and 11 out of 21 tumors (52%), respectively. Based on these results, we may speculate that the biallelic inactivation of the *NF2 *gene is not as important for the development of the meningothelial subtype of meningioma, when compared to the transitional and fibroblastic tumors. A related trend was even more pronounced, when the frequencies of large chromosome 22 deletions were correlated to the three above mentioned histopathological subtypes of meningioma. The presence of large hemizygous deletions showed marked differences between the meningothelial, transitional and fibroblastic subtypes, comprising 13 out of 33 cases (39%), 20 out of 31 cases (65%) and 19 out of 21 cases (90%), respectively. It should be stressed here that 15 out of 19 fibroblastic meningiomas, which displayed gene dosage imbalances on 22q, were monosomy 22 cases. The three remaining tumors (M64, M32, and M114) all showed a minimum loss of 18 Mb of 22q. It may thus be hypothesized that one copy loss of genes located within the 18 Mb on 22q is important for generation of the fibroblastic phenotype of cells.

More than half (61%) of the meningothelial cases were among the category of tumors with both chromosome 22 copies retained. One possible explanation for this finding is that meningothelial tumors more frequently harbor mutations in additional genes that are located outside chromosome 22. Our results correlate well with previous studies by Wellenreuther *et al*. [15] as well as with Evans and colleagues [17] that identified *NF2 *gene mutations in 4/26 (15%) and 1/20 (5%) of meningothelial tumors. The above results from DNA level analysis are supported by analysis of merlin/schwannomin protein expression, which show normal expression in the greater part of meningothelial tumors, but severely reduced expression levels in the majority of fibrous and transitional meningiomas [55-57]. Furthermore, correlation between tumor localization and histological subtype was previously reported. The majority of meningiomas with no chromosome 22 aberrations were localized in the anterior skull base and were predominantly meningothelial [19]. Taken together, these results might suggest that cellular origin plays a role in the genetic pathways which are operative during tumor formation of meningiomas. In the context of our results from chromosome 22, it is interesting to correlate this to the data obtained from chromosome 1, as genetic aberrations affecting these two autosomes are the most frequent genetic abnormalities in meningiomas [31,36]. Furthermore, aberrations of chromosome 1 are also related to recurrence-free survival from the disease [31]. We have recently completed a high resolution analysis of chromosome 1 using the same tumor series [34] and 49 of the samples were studied with similar resolution on both chromosomes (Table 1). There is a strong relationship between changes on both chromosomes, with 67% of samples showing concurrent aberrations. These results clearly indicate the need for a genome-wide and high-resolution array-based characterization of chromosomal aberrations in meningiomas.

## Conclusion

One objective of this study was to identify the maximum number of tumors displaying copy number aberrations of 22q not encompassing the *NF2 *locus. From the category of tumors displaying terminal- and interstitial-deletions we identified at least two minimal overlapping candidate regions outside the *NF2 *locus. These regions allow analysis of a limited number of candidate genes. Interesting candidates in these regions include the *BCR *gene, implicated in chronic myeloid leukemia and acute myeloid leukemia; the *RGR *oncogene involved in RAS-signaling [58] and found to be altered in human T-cell malignancies [42]; and the zinc finger protein encoding gene *ZCWCC1*. One additional candidate region starts at clone ID 390 and extends toward the telomere (Fig 1A). This segment is hemizygously deleted in all but one (M114) of the studied tumors with 22q gene copy number imbalances.

We assessed the point mutation frequency of *NF2 *in combination with array-CGH of 22q. The rationale was to test the hypothesis that mutations also may be located in evolutionarily well conserved non-genic sequences within introns or in the 5' promoter region, in addition to mutations in the exons. We identified 39 point mutations in 100 tumors. In one single case (M22), there were three tumor-specific mutations detected; a point mutation in the CNG *inter1*, a nonsense mutation in exon 13 and monosomy 22. *Inter1 *displays sequence conservation between human and pufferfish, suggesting that it may possess functional properties. However, the fact that the *inter1 *mutation represented one out of three genetic alterations in this tumor, and that we only identified a single mutation in one CNG, suggests that this is not a major mechanism for inactivation of the *NF2 *gene. We also analyzed the methylation status of *NF2 *promoter associated CpGs in order to examine if this mechanism is associated with repressing *NF2 *transcription in sporadic meningiomas. We identified methylation in only one tumor sample (M104), at a single CpG site (Figure 4B). Thus, we conclude that methylation is unlikely to play a major role in silencing of the *NF2 *gene in meningioma.

Profound differences in mutation frequency were observed between various histopathological subtypes of tumors. There was a considerably higher frequency of biallelic *NF2 *inactivation in fibroblastic (52%) compared to meningothelial (18%) tumors. The presence of 22q copy number imbalances were also more common in the fibroblastic (86%) compared to the meningothelial (39%) subtype. Thus, inactivation of *NF2 *combined with the presence of macro-mutation on 22q, might not be as important for the development of the meningothelial subtype, as opposed to the fibroblastic form.

Chromosome 1 genetic abnormalities are frequently observed in meningiomas [31,36]. Forty-nine of the samples studied here were also previously analyzed for gene dosage aberrations on chromosome 1 by array-CGH [34]. We observed a strong correlation between changes on both chromosomes, with 67% of samples showing concurrent aberrations. A genome-wide and high-resolution copy number analysis could contribute to identify additional genetic alterations involved in tumor development, especially for the meningothelial subtype, which does not commonly display *NF2 *and 22q alterations.

## Methods

### Patient samples

Tumor tissue and matched samples of peripheral blood were collected and DNA was isolated using standard methods [59]. Samples were obtained from the Dept. of Neurosurgery, Karolinska Hospital, Stockholm, Sweden. All analyzed tumor samples were reported previously in various studies [7,22,23,31,34,36]. Ethical approval was granted from the Research Ethics Committee, Faculty of Medicine, Uppsala University. Copy number profiling of chromosome 22 was performed on 126 sporadic meningiomas and this sample set comprised 39 meningothelial, 38 transitional, 28 fibroblastic, 9 anaplastic, 6 psammomatous, 1 angiomatous and 5 tumors of unclassified histopathological subtype. For the analysis for point mutations and methylation in the *NF2 *gene promoter, we used a subset of 100 tumor DNA samples (Table 1 and additional file 3).

### DNA copy number profiling using the chromosome 22 genomic microarray

The genomic microarray used to assess DNA copy number status of chromosome 22 has been described previously [37]. In brief, 480 genomic clones derived from the minimal tiling path of chromosome 22, 8 control clones from chromosome X and 30 non-chromosome 22 autosomal control clones were isolated and grown in bacterial cultures overnight. The clone DNA (cosmid, fosmid, BAC and PAC) was purified using Qiagen kits and quantified using the picoGreen assay (Molecular Probes, Inc., Eugene, OR). All clone DNA were verified using STS PCR. The prepared clone DNA stock was amplified by DOP-PCR [60]. A second round of PCR on the DOP-PCR products using an amino linked universal primer was performed and amplification products were printed in quadruplicate on CodeLink™ slides (GE Healthcare, UK). Protocols used for the hybridization and washing procedures have been previously described [37,61]. Image capture and analysis was performed using the axon 4000B Scanner and GenePix 6 image analysis software. For all clones on the array the average, standard deviation and coefficient of variation (CV) for each of the 4 replicas was calculated. Clones displaying a CV above 5% between a minimum of 3 replica spots were discarded from further analysis. Array-CGH profiles are plotted in Microsoft Excel with the clone IDs on the X-axis and the normalized fluorescent ratio intensities on the y-axis. The average fluorescent ratio value for a given set of continuous clones on the array is referred to as the **A**verage **N**ormalized **I**nter **L**ocus **F**luorescence **R**atio or ANILFR value.

### PCR and sequencing

Exons and conserved non-genic sequences (CNGs) in the *NF2 *locus were PCR amplified from genomic DNA derived from one hundred sporadic meningiomas, with customized primers using Ampli Taq Gold (Applied Biosystems) (primer list in Additional file 4). Peripheral blood DNA from phenotypically normal control persons was also PCR amplified and sequenced for CNGs in which mutations were observed. PCR fragments were purified by Exosapit (GE Healthcare, UK) digestion prior to sequencing. Sequencing of amplicons was performed with customized nested primers using ET Terminator (GE Healthcare, UK) and Big Dye v.3.1 (Applied Biosystems) sequencing chemistries, and ethanol precipitated according to standard protocol [59]. Electrophoresis and fluorescent detection was carried out on Megabace 1000 (GE Healthcare, UK) and ABI 3700 (Applied Biosystems) capillary sequencing machines. Processing and analysis of sequencing data was performed using the Staden package [62].

### Methylation profiling by pyrosequencing of bisulfite treated DNA

Pyrosequencing (Biotage, Uppsala, Sweden) was used as method for detection of methylation of cytosines situated 5'of a guanine [53]. Briefly, the DNA was bisulfite treated using EZ-96 DNA methylation kit (Zymo research, Orange, CA, USA) for conversion of unmethylated cytosines into uracils. In the subsequent PCR uracil amplifies as thymidine. Methylated cytosines remain unchanged after treatment. Cytosines not followed by a guanine are not methylated and will therefore be converted to a thymidine after bisulfite treatment and PCR. These thymidines were used as internal controls for the completeness of bisulfite treatment. Human genomic DNA was methylated in vitro by the CpG methylase *M. Sss I *and used as positive controls for detection of methylation. Bisulfite treated DNA were amplified in a 50 μl reaction using Platinum Taq DNA polymerase (Invitrogen Life Technologies, Carlsbad, CA, USA) according to manufacturers protocol. Primers for amplification and sequencing were custom designed using PSQ assay design software (Biotage, Uppsala, Sweden). Data of primers used for amplification and sequencing, as well as the nucleotide sequence analyzed and the dispensation order of nucleotides during sequencing is shown in Additional file 2. One PCR primer in each pair was biotinylated for conversion of the PCR product to a single stranded template. The cycling condition used were: 94°C for 3 minutes, followed by 20 cycles of 94°C for 20 seconds, 65°C minus 0.5°C/cycle for 20 seconds, 72°C for 1 minute, followed by 40 cycles of 94°C for 20 seconds, 55°C for 20 seconds, 72°C for 1 minute and finally 72°C for 10 minutes. 45 μl of PCR product was used for the Pyrosequencing reaction. Sequencing was performed on a PSQ 96MA system with PSQ Gold reagent kit (Biotage, Uppsala, Sweden) according to manufacturer's protocol. Pyro Q-CpG software (Biotage, Uppsala, Sweden) was used for data analysis.

## Authors' contributions

CMH: participated in the overall study design; performed mutation analysis experiments, methylation analysis experiments, array-CGH experiments; analyzed results and drafted the manuscript. PB: participated in the array-CGH study design, performed array-CGH experiments, data analysis and drafted the manuscript. GG: participated in the study design of the mutation screening part and analysis of sequencing data. AP: participated in the methylation analysis study design. ARH: performed mutation screening and analysis of sequencing data. KM: performed printing of the micro-array. CJ: participated in the preparations of the microarray. TM: provided with tumor and blood samples, provided clinical data of all patients. JPD: intellectual input in the overall study design, drafted the manuscript and generated financial support. All authors read and approved the final manuscript.

## Supplementary Material

Additional file 1 Table summarizing DNA copy number changes in the additional subset of 26 sporadic meningiomas not studied for NF2 gene point mutations or NF2 promoter methylation status. Click here for file

Additional file 2 Table summarizing primers used for PCR amplification and sequencing of the NF2 gene. Click here for file

Additional file 3 Table summarizing primer pairs used for amplification of bisulfite treated DNA, sequencing primers, analyzed sequences and dispensation order. Click here for file

Additional file 4 List of non-22q-derived genomic clones that encompass genes of potential interest in tumor formation/progression. Click here for file

## Figures and Tables

**Table 1:** Genetic alterations in one hundred meningiomas.

Sample ID	Subtype^1^	CH_3_	NF2 gene mutations			DNA copy number profiles^4^		
			Position	Mutation^2^	Assumed effect^3^	Chromosome 22	Chromosome 1	Loss of non-22 genes^5^
M26	A		Ex 2	227_240del	Lys76fs	Monosomy	Monosomy	D1, S1A, S1B
M71	A		Ex8	810+2T>C	Abn. Spl.	Monosomy	1p, 120.8 Mb TD	D1, P2A, S1A, S1B
M38	A		Ex6	573del; 575_581del	Trp191fs	Monosomy	1p 107.9 Mb TD	P1B, P2A, S1A, S1B
M43	A		Ex12	1185del	Lys396fs	Monosomy	1p 120.3 Mb TD, 4.4 Mb A	
M46	A		Ex8	810+2T>C	Abn. Spl.	NAD	NAD	
M33	A			NAD		30.6 Mb TD [AC009516-AC002055]	1p, 90.2 Mb TD, 45.8 Mb ID	
M9	A			NAD		NAD	NAD	
M20	A			NAD		NAD	NAD	
M13	F		Ex 2	240+2T>C	Abn. Spl.	Monosomy	1p, 142.9 Mb TD	P2A, S1A, S1B
M88	F		Ex4	364-1G>A	Abn. Spl.	Monosomy	1p 84.1 Mb TD; 1q 4.6, 7.1 Mb IDs, 12.5 Mb TD	P2A
M3	F		Ex 2	240+2T>C	Abn. Spl.	Monosomy	1p 119.8 Mb TD	
M14	F		Ex3	249del	His84fs	Monosomy	1p 120.2 Mb TD	
M15	F		Ex8	683del	Lys228fs	Monosomy	1p 116.0 Mb TD	
M39	F		Ex8	810+1G>A	Abn. Spl.	Monosomy	NS	
M42	F		Ex10	995del	Lys332fs	Monosomy	NS	
M70	F		Ex6	599+1G>A	Abn. Spl.	Monosomy	NS	
M91	F		Ex 2	154del	Arg52fs	Monosomy	NS	
M102	F		Ex9	865A>T	Lys289X	Monosomy	NS	
M115	F		Ex7	634C>T	Gln212X	NAD	1p 119.6 Mb TD	
M64^6^	F			NAD		30.4 Mb TD [AC008079-AC002055], *NF2 *biallelic del [AC005529]	1p 98.8 Mb ID; 1q 24.2 Mb ID	P1B, P2A
M66	F			NAD		Monosomy	1p 118.1 Mb TD	P2A
M11^7^	F			NAD		0.55, 2.8, 4.5 Mb IDs [U07000-AP00034, AC005004-Z69907, AL022311-Z83851]; 5.9 Mb TD [Z93244-AC002055]	1p 116.8 Mb TD, 3.6 Mb A	
M32	F			NAD		5.3 Mb ID [AL022329-AL022336], 15.2 Mb TD [AL009049-AC002055]	NS	D1
M4	F			NAD		Monosomy	NAD	
M29	F			NAD		Monosomy	NS	
M41	F			NAD		Monosomy	NS	
M92	F			NAD		Monosomy	NS	
M114	F			NAD		18.0 Mb ID [AP000525-Z68324]	NS	
M119	F			NAD		NAD	NS	
M10	M		Ex13	1341-2A>G	Abn. Spl.	Monosomy	1p 120.3 Mb TD	P2A
M23	M		Ex3	241-2A>C	Abn. Spl.	Monosomy	1p 120.9 Mb TD	P2A
M67	M		Ex4	432C>A	Tyr144X	Monosomy	1p 120.8 Mb TD	P2A
M98	M		Ex14	1519_1520del	Phe507fs	Monosomy	NAD	D1, P2A, S1A, S1B
M57	M		Ex 2	133_144 del	Asp45fs	Monosomy	NS	
M72	M		Ex7	634C>T	Gln212X	Monosomy	NS	
M40	M			NAD		Monosomy	1p 164.9 Mb TD	P1B, P2A
M76	M			NAD		Monosomy	1p 142.7 Mb TD	P2A
M105	M			NAD		Monosomy	1p 116.3 Mb TD	P1B, P2A
M34^8^	M			NAD		20.0 Mb TD [AC004542-AC002055]	1p 102.8 Mb ID	
M44	M			NAD		Monosomy	1p 85.4 Mb TD	
M101	M			NAD		25.1 Mb ID [AP000525-AL035450], 7.9 Mb TD [AL049758-AC002055]	Monosomy, 1p 2.1 Mb R	
M30	M			NAD		Monosomy	NAD	D1, P2A
M112	M			NAD		NAD	1p 114.2 Mb TD	
M2	M			NAD		NAD	NAD	
M5	M			NAD		NAD	NAD	
M77	M			NAD		NAD	NAD	
M82	M			NAD		NAD	NAD	
M45	M			NAD		NAD	NS	
M51	M			NAD		NAD	NS	
M54	M			NAD		NAD	NS	
M61	M			NAD		NAD	NS	
M63	M			NAD		NAD	NS	
M65	M			NAD		NAD	NS	
M69	M			NAD		NAD	NS	
M86	M			NAD		NAD	NS	
M95	M			NAD		NAD	NS	
M99	M			NAD		NAD	NS	
M107	M			NAD		NAD	NS	
M108	M			NAD		NAD	NS	
M111	M			NAD		NAD	NS	
M116	M			NAD		NAD	NS	
M120	M			NAD		NAD	NS	
M93	P		Ex8	810G>T	Glu270Asp	Monosomy	1p 114.3 Mb ID	P2A
M22^9^	P		Ex13, inter1	1390del; 28309611 C>A	Ala464fs, unknown	Monosomy	NS	
M62	P			NAD		Monosomy	NS	
M21	P			NAD		NAD	NS	
M60	T		Ex14	1519_1528del	Phe507fs	Monosomy	1p 118.2 Mb TD	P2A
M1	T		Ex7	650del	Tyr217X	Monosomy	1p 65.0 Mb TD	
M31	T		Ex13	1325del	Ala451fs	Monosomy	1p 53.2, 36.3 Mb IDs	
M58	T		Ex 2	115-3G>C	Abn. Spl.	30.1 Mb TD [AC007050-AC002055]	1p 120.2 Mb TD	
M90	T		Ex3	241-2A>G	Abn. Spl.	20.1Mb ID [AP000525-AL079295], 13.4 Mb TD [AL049850-AC002055]	1p 83.3 Mb TD	
M96	T		Ex12	1175G>T	Glu392X	2.0 Mb ID [AP000525-AC007666], 5.4 Mb A [AC006285-AC000102], 27.5 Mb TD [U07000-AC002055]	1p 43.3 Mb TD, 1q 69.9 Mb TD	
M52	T		Ex4	431_432insAA	Tyr144X	Monosomy	NS	P1B
M113	T		Ex4	377del	Leu127X	Monosomy	NAD	D1, P1B, P2A, S1A, S1B
M18	T		Ex12	1198C>T	Gln400X	Monosomy	NS	
M37	T		Ex8	745_755del	Arg249fs	Monosomy	NS	
M48	T		Ex 1	36_37del	Leu14fs	Monosomy	NS	
M75	T		Ex8	750del	Thr251fs	Monosomy	NS	
M87	T		Ex4	436G>A	Val146Ile	NAD	NS	
M56	T			NAD		Monosomy	1p 116.5 Mb TD	P2A
M85	T			NAD		Monosomy	1p 108.2 Mb TD	P2A
M109	T			NAD		5.7 Mb A [AC006548-AC007957], 30.6 Mb TD [AC009516-AC002055]	1p 120.2 Mb TD	
M27	T			NAD		15.5Mb ID [AP000525-AC005005], 14.1 Mb TD [AL049749-AC002055]	NS	
M59	T			NAD		Monosomy	NS	
M68	T			NAD		Monosomy	NS	
M94	T			NAD		Monosomy	NS	
M97	T			NAD		Monosomy	NS	
M25	T			NAD		NAD	1p 98.7 Mb TD	
M117	T			NAD		NAD	1p 36.1 Mb TD	
M104^10^	T	+		NAD		NAD	NS	
M8	T			NAD		NAD	NS	
M12	T			NAD		NAD	NS	
M17	T			NAD		NAD	NS	
M35	T			NAD		NAD	NS	
M55	T			NAD		NAD	NS	
M89	T			NAD		NAD	NS	
M103	T			NAD		NAD	NS	
M78	U		Ex10	999+1G>T	Abn. Spl.	Monosomy	1p 3.8 Mb TD	
M79	U			NAD		NAD	1p 39.1Mb ID, 1q 41.3Mb TD	
M83	U			NAD		NAD	NS	

**1 **Tumors are grouped according to histopathological subtypes. Abbreviations: A, Anaplastic; F, Fibroblastic; M, Meningothelial; P, Psammomatous; T, Transitional and U, Unclassified.

**2 **Position of the mutations according to numbering of the cDNA sequence, nucleotide number one is the A in the ATG translation initiation codon [GenBank:AF369658]. Abbreviations: substitution, >; deletion, del; insertion, ins; No Aberration Detected, NAD. Donor splice site mutations indicated by the last nucleotide of the preceding exon, a plus sign and position in the intron (e.g. 240+2T>C). Acceptor splice site mutations denoted by the first nucleotide of the following exon, a minus sign and position in the intron (e.g. 241-2A>C).

**3 **Amino acid number one is the translation initiator Methionine. Abbreviations: translation termination codon, X; frame shift, fs; abnormal splicing, Abn. Spl.

**4 **Data from copy number imbalances on chromosome 1 were previously published [34]. Abbreviations: Terminal Deletion, TD; Interstitial Deletion, ID; Amplification/gain, A; Not Studied, NS; No Aberration Detected, NAD; Retained segment R. Genbank accession numbers in squared brackets.

**5 **Loss of non-chromosome 22 clones spanning the *DAL1*, D1; *PTCH2A*, P2A; *PTCH1B*, P1B; *SUFU1A*, S1A; and *SUFU1B*, S1B genes.

**6 **M64 displays a homozygous deletion of the *NF2 *gene.

**7 **M11 demonstrates multiple deletions not affecting the *NF2 *gene.

**8 **M34 displays a terminal deletion not affecting the *NF2 *locus.

**9 **M22 presents two mutations in the *NF2 *locus, in exon 13 and in the CNG *inter1*. The position of the *inter1 *mutation is according to numbering on chromosome 22 (UCSC Homo sapiens Genome Browser, assembly July 2003). Ten bases on each side of the mutation: agcaaagagaCaaattaccca.

**10 **Methylation of a single CpG site in the intron 1 of the *NF2 *gene in ~40% of DNA molecules.

**Table 2:** Chromosome 22 DNA copy number status and *NF2 *gene mutations in a subset of 100 sporadic meningiomas.

	***NF2 *gene**		
		
**Chr. 22 status**	**Point mutation**	**No point mutation**	**Total**
Monosomy	32	17	49
NAD^1^	3	36	39
Interstitial/terminal deletion	3	9	12
Total	38	62	100

**1 **No Aberration Detected, NAD

**Table 3:** *NF2 *gene mutation frequencies in meningiomas of different histological subtypes.

**Histological subtype**	**Total no.**	***NF2 *gene mutation frequency^1^**	**Chr. 22 deletions affecting the *NF2 *gene**	**Biallelic inactivation of the *NF2 *gene**
Meningothelial	33	6/33 (18%)	13/33 (39%)	6/33 (18%)
Transitional	31	13/31 (42%)	20/31(65%)	12/31(39%)
Fibroblastic	21	11/21 (52%)	18/21 (86%)^4^	11/21 (52%)^5^
Anaplastic	8	5/8 (61%)^2^	5/8 (61%)	4/8(50%)
Psammomatous	4	2/4 (50%)^3^	3/4 (75%)	2/4 (50%)
Unclassified	3	1/3 (33%)	1/3 (33%)	1/3 (33%)

**Overall**	100	39	60	36

**1 **The methylation status of *NF2 *gene promoter in tumor M104 is not included.

**2 **The two micro deletions (1 bp and 7 bp) detected in M169 within exon 12 are considered as one.

**3 **Two point mutations were detected in M22, only one is used for calculations.

**4 **Fifteen of the 18 fibroblastic meningiomas that display deletions on chromosome 22 are monosomy cases. The three remaining tumors (M64, M32, and M114) all disclose a minimum of 18 Mb deletion of 22q.

**5 **Case M64 (biallelic deletion affecting the *NF2 *gene) is not included in this calculation
