# Supplementary material for: Comprehensive genetic and epigenetic analysis of sporadic meningioma for macro-mutations on 22q and micro-mutations within the NF2 locus
Source: BMC Genomics. 2007 Jan 12;8:16. doi: 10.1186/1471-2164-8-16 (PMC1781436; doi:10.1186/1471-2164-8-16)
Supplement: Additional file 1 — Table summarizing DNA copy number changes in the additional subset of 26 sporadic meningiomas not studied for NF2 gene point mutations or NF2 promoter methylation status. [file 1471-2164-8-16-S1.pdf]

Additional file 1. List of non- 22q-derived genomic clones that encompass genes of potential interest in tumor formation/progression.

Clone name	Gene
RP11-286N3	*DAL-1*
RP11-78G22	*DAL-1*
RP11-34D4	*PTCH1B*
RP11-102H4	*PTCH2A*
RP11-47A8	*SUFU1A*
RP11-170J3	*SUFU1B*
