# Supplementary material for: Comprehensive genetic and epigenetic analysis of sporadic meningioma for macro-mutations on 22q and micro-mutations within the NF2 locus
Source: BMC Genomics. 2007 Jan 12;8:16. doi: 10.1186/1471-2164-8-16 (PMC1781436; doi:10.1186/1471-2164-8-16)
Supplement: Additional file 2 — Table summarizing primers used for PCR amplification and sequencing of the NF2 gene. [file 1471-2164-8-16-S2.pdf]

Additional file 2. Table summarizing primer pairs used for amplification of bisulfite treated DNA, sequencing primers, analyzed sequences and dispensation order.

PCR primer pair	NF2_F1	TGGTGGAGTTATTTTAAAGGAGG
	NF2_R1	CACAAACCTCAAAACCACCATAAT
PCR product size	550 bp	
Sequencing primer	NF2_S1c	ATTGGTGGAGTTATTTTAAAGGAG
Sequence to analyze		GYGGGAYGGAGYGGGAAAGTTTTGTTTATTTTGGTTTTTTTAGTTAATYGTAGTATTTATGYGTTAGTTTTYGGTTYGG
No of CpGs	7	
No of bases	79	
Dispensation order	60	AGTCGTATCGATGTCGAGTTGTATTGTTAGTGATCGTAGTATATAGTCGTATGTTCAGTC
Sequencing primer	NF2_S2	GGAGTGAGGAGGGTGATA
Sequence to analyze	9	GTTAYGYGYGYGYGYGTAYGYGTTYGATGTAGYG
No of CpGs	9	
No of bases	32	
Dispensation order	47	TGTGATCTGTCAGTCTGTCAGTCGTGATCTGTCAGTCGATGTATGTC
Sequencing primer	NF2_S3	GGGTTGTGGGGAGGG
Sequence to analyze		TGYGTTTTTYGYGGGYGYGYGGAGTGAGGAYG
No of CpGs	7	
No of bases	32	
Dispensation order	46	GTAGTCTGTTCAGTCTGTCAGTCTGTCGAGTGAGTATCGTGATAGT
Sequencing primer	NF2_S5	AGTGAGGAAGGTGATAGTTA
Sequence to analyze		YGYGYGYGYGTAYGYGTTYGATGTAGYGYGGTTTYG
No of CpGs	11	
No of bases	36	
Dispensation order	52	ATCTGTCAGTCTGTCAGTCGTGATCTGTCAGTCGATGTATGTCAGTCTGTTC
PCR primer pair	NF2_F7	GGTGGTTTTGAGGTTTGTGTAGTA
	NF2_R7	AAAACATCCAACTTCTTCCAATAA
PCR product size	339 bp	
Sequencing primer	NF2_S8	GGAGTTTAATTGAGAGGTAA
Sequence to analyze		TYGGTYGGTAGTTTYGATTGTTGYGGTGATAGTYGAGGTGGAAGTTYG
No of CpGs	6	
No of bases	48	
Dispensation order	46	ATCTGTCGTATGTTCGATGTAGTCGTGATATGTCGAGTGATGTCGA
