# Supplementary material for: Comprehensive genetic and epigenetic analysis of sporadic meningioma for macro-mutations on 22q and micro-mutations within the NF2 locus
Source: BMC Genomics. 2007 Jan 12;8:16. doi: 10.1186/1471-2164-8-16 (PMC1781436; doi:10.1186/1471-2164-8-16)
Supplement: Additional file 3 — Table summarizing primer pairs used for amplification of bisulfite treated DNA, sequencing primers, analyzed sequences and dispensation order. [file 1471-2164-8-16-S3.pdf]

Additional file 3. Table summarizing DNA copy number changes in the additional subset of 26 sporadic meningiomas not studied for *NF2* gene point mutations or *NF2* promoter methylation status.

Sample ID	Subtype[1]	DNA copy number profiles[2]	
		Chromosome 22	Chromosome 1
M157	A	NAD	TD 1p (1-1185)
M100	Ang	Monosomy	TD 1p (1-1100)
M84	F	NAD	TD 1p (1-33)
M136	F	NAD	TD 1p (1-840)
M145	F	Terminal deletion	TD 1p (1-898)
M149	F	NAD	TD 1p (1-762)
M155	F	TD from clone 167	TD 1p and ID
M166	F	NAD	TD 1p and ID
M172	F	Monosomy	NS
M134	M	Monosomy	TD 1p, 2 levels of fluorescent ratio loss (1-1159) (1160-1379)
M150	M	NAD	TD 1p (1-1159)
M151	M	Monosomy	NAD
M161	M	Monosomy	TD 1p and ID
M169	M	Monosomy	NS
M173	M	NAD	NS
M174	M	NAD	NS
M106	P	Terminal del	1p ID
M165	P	NAD	NS
M36	T	Monosomy	NS
M47	T	Monosomy	NS
M121	T	Monosomy	TD 1p (1-681)
M137	T	NAD	NAD
M156	T	TD from clone 91	TD 1p and ID
M159	T	NAD	TD 1p and A
M162	T	Monosomy	TD 1p (1-1010)
M168	U	NAD	TD 1p (1-1041)

1 Tumors are grouped according to histopathological subtypes. Abbreviations: Anaplastic, A; Angiomatous, Ang; Fibroblastic, F; Meningothelial, M; Psammomatous, P; Transitional, T; and Unclassified, U.

2 Data from copy number imbalances on chromosome 1 were previously published and naming of tumor samples is identical [34]. Abbreviations: Terminal Deletion, TD; Interstitial Deletion, ID; Amplification/gain, A; and Not Studied, NS.
