# Supplementary material for: Comprehensive genetic and epigenetic analysis of sporadic meningioma for macro-mutations on 22q and micro-mutations within the NF2 locus
Source: BMC Genomics. 2007 Jan 12;8:16. doi: 10.1186/1471-2164-8-16 (PMC1781436; doi:10.1186/1471-2164-8-16)
Supplement: Additional file 4 — List of non-22q-derived genomic clones that encompass genes of potential interest in tumor formation/progression. [file 1471-2164-8-16-S4.pdf]

Additional file 4. Table summarizing primers used for PCR amplification and sequencing of the *NF2* gene

PCR-primer no.	Primer name	Sequence	Tm	PCR product (bp)
1	jd.Inter1.p1.U	GGCTGGCTAGCACAATTAG	60.7	695
2	jd.Inter1.p1.L	GTTTTTATGTGGCACTCTGTC	59.5	
3	jd.Inter2.p1.U	CCAGCCTTTCTGGACTAT	56.4	1336
4	jd.Inter2.p1.L	GCTTGAATGATGTTGTTGTAT	57.3	
5	jd.Inter3_4.p1.U	GTCCTCCCAGGAACATTA	56.4	825
6	jd.Inter3_4.p1.L	TCAGGAAATGTGAATTACAG	55.1	
13	jd.int1.p2inner.U	CATAGTCCCTGGCAGAAA	57.9	1044
14	jd.int1.p2inner.L	CTTACTTCTGAAACAGGAATAG	56.1	
67	jd.ex1.p3.U	AACCCAAATTCAGCAATT	57.1	2103
68	jd.ex1.p3.L	CCACCTTGCCATTTACTA	55.8	
69	jd.ex1.p4.U	ATGGGCATCATAGAGTAT	50.6	2186
70	jd.ex1.p4.L	GGCTGTGAGCTTGGTTC	57.5	
171	jd.ex1.p5.U	CCCTACACCCGTTAGAA	54.9	2247
173	jd.ex1.p5.L	TTGAGAAGCAGTACTGTACG	55.6	
172	jd.ex1.p6.U	CCGTTAGAACCCTTGATGAC	61.7	2199
15	jd.ex2.p1.U	CCATGTCTGTCGAAAGAT	54.1	1385
16	jd.ex2.p1.L	ATCAGGCTGGTAACAATG	54.8	
153	jd.ex3.p2.U	AAAGGCAGTGGTTTACAGAC	59.0	2046
154	jd.ex3.p2.L	GGGACAAACCAAGTGTCAC	59.4	
19	jd.ex4up1.p1.U	TGTTCTTGCAGTTTTTCTATAG	57.6	896
20	jd.ex4up1.p1.L	ACAGGTGGAGAGTTCAGTC	55.0	
21	jd.Ex4.p1.U	GGGCTGAGGCTGTAGTCAT	61.7	1123
22	jd.Ex4.p1.L	ACCCTCTCATCGGTCTTGT	60.6	
23	jd.Int4.p1.U	ATTGGCTCCACAAGTCTTTTA	61.8	686
24	jd.Int4.p1.L	GCCATCCTCTGCATTACTT	59.5	
25	jd.Ex5.p1.U	AGGCTCTTTGAGAATGAGAG	58.1	1216
26	jd.Ex5.p1.L	TGGTGACCCCAAACTAACT	59.4	
27	jd.Ex6.p1.U	GGGCATTGTGGTTTTTAAT	59.9	810
28	jd.Ex6.p1.L	ACAGGCAGTACACATACCTACT	58.3	
147	jd.Ex.7.p3.L	AATGCTTTGGCAAATCG	59.3	810
148	jd.Ex.7.p3.U	TCTGGCAGCGACGTG	57.8	
31	jd.Ex8.p1.U	TATGGGCTTTAATTCTATACC	56.8	943
32	jd.Ex8.p1.L	GCTCAATAAAGACCACACTTA	57.2	
33	jd.Ex9.p1.U	CCAGGACAAGGCATAACTT	59.5	788
34	jd.Ex9.p1.L	GTGGGTACACAGCTATTCACT	58.9	
35	jd.Ex10.p1.U	GCTTTGGTGTTTCACTCTATG	59.8	1179
36	jd.Ex10.p1.L	GCCACGAGCTCTTCATG	58.6	
37	jd.Int10.p1.U	CAGGAACTGAAGGAGATTAC	55.7	1641
38	jd.Int10.p1.L	GCCACTTGAAAAACAGTCTA	57.5	
39	jd.Ex11.p1.U	GGGGTCCATGTGGATTAG	59.4	998
40	jd.Ex11.p1.L	CAAACGCTGCTAACTCAT	55.9	
41	jd.Ex12.p1.U	GAGAAGGTGCTCTTGTTACTC	58.0	1085
42	jd.Ex12.p1.L	TAAAAGCCAAATTCAAGTG	56.1	
43	jd.Ex13.p1.U	TCACATGCCGTGACCA	57,6	645
44	jd.Ex13.p1.L	GGCTCACCGGGTACG	58,6	
45	jd.Ex13d1.p1.U	AAGCCCACGTACCCG	58,6	1021
46	jd.Ex13d1.p1.L	CGGAGAACCGGGATATA	57,8	
47	jd.Ex14.p1.U	CCCAAGCTCCTAATCCG	60,4	776
48	jd.Ex14.p1.L	GGGCCCTCGCTTAGAT	59,5	
49	jd.Ex15.p1.U	TCAAACCCTAGATCGCA	56.0	1096

50	jd.Ex15.p1.L	CCCTGGAGTTTTCAGATAC	56.0	
51	jd.Ex16.p1.U	GGAGGCCTCGGTCATTA	60,2	1919
52	jd.Ex16.p1.L	GGAGCAGTGCAAATTTAACTA	60,3	
179	jd.ex17.p2.U	CAGGGCATGAGGACTAA	55.1	993
180	jd.ex17.p2.L	GGGCTCCCTGACGATGA	63.2	
181	jd.ex17.p3.U	CTCAGGGACCTTGGTAGAT	57.9	929
182	jd.ex17.p3.L	ACCCAGCACTTGCTCGTC	61.7	

**Sequencing primer no.**	**Primer name**	**Sequence**	**Tm**
73	jd.inter1.s1.U	TTAGGGTTGGACTCAGTTCT	58.3
74	jd.inter1.s2.U	GGCAGGTTCATAAGTGTAAA	57.6
75	jd.inter2.s1.U	CCACTCCGCGTATAGATTA	58.4
169	jd.inter2.s1.L	TAGCCTTTTAAAGTGGTTTATAA	59.6
169	jd.inter2.s1.L	TAGCCTTTTAAAGTGGTTTATAA	59,6
175	jd.inter2.s2.L	CCCAAGCACGTCAACAA	59,9
176	jd.inter2.s3.L	GGGCATTTGCTTCCTTG	61,1
170	jd.inter3.s2.L	TGCGTGACACTGGGAAC	59.1
76	jd.inter3.s1.U	CATTAAATTTGGCATTTGG	59.1
77	jd.inter4.s1.L	GCAGACATTGTTGCTGTAAC	58.2
78	jd.int1.s2.U	AGGTGGCTTTTCAAGAAG	56.8
79	jd.int1.s3.U	GCCTCCTTCTCCTGTTGT	58.4
80	jd.int1.s3.L	AAAGCCACCTACATTTGAC	56.8
192	jd.ex1.s1.U	AGTGCCAGCAAACCTAACA	60.9
193	jd.ex1.s2.U	GAGTGAGGACGGTGACAG	56.7
194	jd.ex1.s3.U	GCCCCGTGACCCTAGTC	62.1
195	jd.ex1.s4.U	GGCCTGTGCAGCAACTC	61.4
196	jd.ex1.s5.U	TTCCCGCATGAGCTTCA	62.7
197	jd.ex1.s1.L	GAAAAGGGAAACAAGTATTG	56.7
198	jd.ex1.s2.L	AAGACGCAACTTCTAATACC	56.4
199	jd.ex1.s3.L	TAAGCGTAAGCCTAACTTAA	56.1
200	jd.ex1.s4.L	TTAAGCCTGACACTGAAATC	57.3
201	jd.ex1.s5.L	TGCCAAAACCCAAAAGTC	60.9
202	jd.ex1.s6.L	AAAGCAGCAATCATCTTTA	55.5
203	jd.ex1.s7.L	CAAAACCCAAAAGTCCTATGA	62.1
204	jd.ex1.s8.L	TGTCACCGTCCTCACTC	54.2
205	jd.ex1.s9.L	GTCGCCCAGACCACTAC	57.1
206	jd.ex1.s10.L	GGAGGCTGACGCATAGAT	59.7
207	jd.ex1.s6.U	ACGCCGAGATGGAGTTC	59.9
208	jd.ex1.s7.U	CCCCAACTCCCCTTTC	59.1
209	jd.ex1.s8.U	TCCCCTTTCCGCTCAG	60.1
210	jd.ex1.s9.U	AAAGGGCTCAGAGTGC	53.8
215	jd.ex1.s10.U	ACTGCCAAAGAAGAATATTC	56.1
216	jd.ex1.s11.U	ACACTCGGAGCCTACTTGA	59.6
217	jd.ex1.s12.U	ATAGGGGTCGGTATATGTGA	59.1
218	jd.ex1.s13.U	GCCTCCACAAGTGTCTTTA	57.4
219	jd.ex1.s14.U	GCCTCAACCAGTCTTGTTA	57.4
220	jd.ex1.s11.L	GGTTGCTTCCTCTTGAGAG	58.8
221	jd.ex1.s12.L	CTTCCACCTCGACTGTCAC	59.5
222	jd.ex1.s13.L	ACGGCCTGCACTCTGAG	60.9
223	jd.ex1.s14.L	TGCACTCTGAGCCCTTTAG	60.6
224	jd.ex1.s15.L	GAGGACCCTGCCTGAGC	62.1
82	jd.ex2.s2.U	TTTCAAGGGAATGAATGTC	57.2
83	jd.ex2.s3.U	CCCCATTGGTTTGTTATT	57.4
177	jd.ex2.s4.L	TCTCCAAAGCTGCTAATTTAG	60
213	jd.ex2.s4.U	CCCCGAGTTAACTTAAGAG	56.6

214	jd.ex2.s5.U	ACCAGCTTTCCTAAATTAGC	58.7
84	jd.ex3.s1.U	ATAGGTGACTTTGTGAATACTTC	57.7
85	jd.ex3.s2.U	TACCCCCCAGTTCTGAGA	59.6
185	jd.ex3.s3.U	TCTTGGGGCTATTGATTAA	58
186	jd.ex3.s2.L	AGAGCAAAAGACAAACACAC	56,9
190	jd.ex4.s5.L	AACAGTGACAGTGGTTCTT	
191	jd.ex4.s6.L	CACAAGTCCCATAACATCTCA	
87	jd.ex4up1.s1.U	ACTGCCTGTCTCTTGTGTGA	60.0
88	jd.ex4up1.s2.U	GCTTCCTTTTCCCTTAGAG	58.3
89	jd.ex4up1.s1.L	CTCCTCTCACAGATGTCAACA	59.6
92	jd.ex4.s3.U	TTCCTGTGTAATCTCATTATGT	56.6
178	jd.ex4.s4.U	GTTGCTCCTTTCAGGTAA	54,6
93	jd.int4.s1.L	GAAAGGGTAGGATGCTAAA	56.8
95	jd.ex5.s2.U	ATGGCAGTGTTGACATCAG	58.0
96	jd.ex5.s3.U	TCACCCATCCATCTCTTAC	56.4
155	jd.ex5.s4.U	CTGTTGGTGGCTAAAAGA	55.6
158	jd.ex5.s1.L	AATTGGAGCCCATTACTAG	56,7
97	jd.ex6.s1.U	CCCTCTCTCTGTGTGACTAT	55.1
98	jd.ex6.s1.U	GGTATTGTTTGCCACTTTT	57.3
159	jd.ex7.s1.L	CAGAGCCAGAAATAATCA	52.5
160	jd.ex7.s2.U	TCCACCCATCTCACTTAG	54.2
161	jd.ex7.s3.U	CAAGGCGATAGACTCTTT	54.1
162	jd.ex7.s4.U	GCTCCTTGTGTAGTAGACAG	53.8
163	jd.ex7.s5.L	CTTCCTCTTTCCTTCTTTAC	55.2
99	jd.Ex8.s1.L	TGGGGGTCAGTCTGTTCT	59.0
100	jd.Ex8.s1.U	TGGGACCTGCTGAAACT	57.3
101	jd.Ex8.s2.U	TGACAAGGAGGTAGGACAT	56.1
102	jd.Ex9.s1.U	GACAAGGCATAACTTCATG	55.0
103	jd.Ex9.s1.L	TCATGATATAATTTAGTTTT	47.0
104	jd.Ex10.s1.U	GCATTCATCTTCACGTTTAC	57.6
105	jd.Ex10.s2.U	GCCGTGGACATACCTGCT	63.1
106	jd.Ex10.s3.U	GCTGGAATGCTAATTTGACT	59.5
107	jd.int10.s1.U	CAAGCAATAAAACAGTGAAAG	58.2
108	jd.int10.s2.U	AGCCACCTTATGGAAATTGT	61.2
109	jd.int10.s3.U	AAGCAGCAGTCTTAGAAACC	58.8
110	jd.int10.s4.U	GCTCCCCCCTGTGAAAT	61.7
111	jd.ex11.s1.L	GCCATTGTTGCTTCTTCTT	59.8
114	jd.ex12.s2.U	CTGCTTGCCCATCTTCT	57.5
168	jd.ex12.s2.L	CCTCAGCCATCTTCAGTG	57,7
183	jd.ex12.s3.U	GGAGGAGGCAAAACTTCT	58,3
184	jd.ex12.s3.L	AAGCGGTCTGTGGATATG	57,7
115	jd.ex13.s1.U	TTCCTGCTCTTCCAGTTT	56.8
116	jd.ex13d1.s1.L	GCACGGCAGTAATTCACTC	60.6
117	jd.ex13d1.s1.U	CTGAGCTGAATGTAATCTTC	54.1
118	jd.ex14.s1.L	ACAACCCAGTCCTACACTTT	58.0
119	jd.ex14.s1.U	AGGTTTGCTCTGCTCTTAC	56.4
164	jd.ex14.s2.L	GCCCCAATCACTCAGTCTAGT	63
174	jd.ex14.s3.L	TGAATGCTGATCTGTTGTC	55,2
120	jd.ex15.s1.L	TCCCTTTCTGGCATAAGTT	59.4
121	jd.ex15.s1.U	TGGCAGCAGCAAGCACA	64.7
123	jd.ex15.s2.U	TTTCCCTCTAGTCTTGTGG	56.6
211	jd.ex15.s3.U	AGCCGTGTCTCACTGTCT	56.3
212	jd.ex15.s2.L	ACAGGGGTTCATTACATCA	57.1
124	jd.ex16.s1.L	CCTGGGAACAACCTACTTT	58.6
125	jd.ex16.s1.U	TGGCACTTATGGCATTGT	59.2
126	jd.ex16.s2.U	AGGACCAGCCATTCTTC	56.0

127	jd.ex16.s3.U	GCAAACCCAGAGCACAG	58.5
128	jd.ex16.s4.U	CAGCAGCATAGGATGTGTC	58.2
129	jd.ex16.s5.U	ATCCTTGTTGGCATTGATA	57.9
188	jd.ex17.s2.U	TCCCAGCCACCTTTGAG	60,9
189	jd.ex17.s1.L	AGGTCAGCTCTGGAGTGC	59,1
